# Recreational MDMA use in Norway: results from an internet convenience sample

**DOI:** 10.3389/fpsyt.2025.1619676

**Published:** 2025-09-26

**Authors:** Cato Grønnerød, Thea Rønningen, Ingrid Rike Haugsjå, Kristoffer A. A. Andersen, Camilla Lindvall Dahlgren, Tor-Morten Kvam

**Affiliations:** ^1^ Department of Psychology, University of Oslo, Oslo, Norway; ^2^ Section for Clinical Addiction Research, Oslo University Hospital, Oslo, Norway; ^3^ Department of Psychology, Oslo New University College, Oslo, Norway; ^4^ Faculty of Medicine, University of Oslo, Oslo, Norway; ^5^ Nordre Østfold DPS, Østfold Hospital Trust, Grålum, Norway

**Keywords:** MDMA, 3,4-methylenedioxymethamphetamine, survey, recreational use, mental health, mental disorders, substance use disorders, drug use

## Abstract

**The purpose of the article:**

In recent years, a renewed interest has emerged in investigating the use of MDMA in the treatment of mental disorders. However, knowledge about the characteristics of recreational use of MDMA, its contexts and effects are limited.

**Methods:**

We recruited adult Norwegian participants aged 18 to 65 who reported having had a memorable experience after using MDMA. They completed an anonymous internet survey with 150 items covering matters related to recreational use of MDMA, as well as four standardized measures related to their experience and to personality functioning. We present descriptive statistics (frequencies, means, and standard deviations) from the survey.

**Results:**

We recruited 654 participants, 608 of which were eligible for inclusion in the data analysis (60.5% male; 89% 45 years or younger). Participants reported recreational (65.5%) and therapeutic (22.9%) motivation for MDMA use, mostly at home (28.3%) or at somebody else’s home (34.4%). Participants were well prepared (63%) and most had a clear intention behind their use (54.3%). They were clearly in favor of using MDMA therapeutically (84.7%). Mental distress or disorders were frequently reported (82.1%), but also a large degree of improvement, especially for internalizing disorders such as PTSD, social anxiety and depression. Persistent negative effects were relatively rare and short lived.

**Conclusions:**

Our sample reported positive experiences and effects of recreational MDMA use, with a small minority reporting problematic effects and negative experiences. Self-perceived symptoms were reported as improved, especially for internalizing disorders. Participants reported positive changes in many aspects of life.

## Introduction

MDMA remains widely used recreationally, despite being classified as a Schedule I substance in the United States and a Class A drug in the United Kingdom. In the US, a representative study of individuals aged 12 and older (N = 315,661; 2015–2020) estimated past-year use at 0.9% (1). In the EU, past-year MDMA use among young people was reported at 1.4% (2). Indications of MDMA use in Norway have varied across studies. In 2014, 2.3% of 15–34-year-olds reported lifetime ecstasy/MDMA use (3). By 2024, lifetime use had increased considerably, with 6.5% of individuals aged 16–64 reporting use at some point, and 1.4% reporting use in the past year (4).

Epidemiological data on MDMA use is crucial for informing public health policies and harm reduction strategies. However, comprehensive data on its use specifically, especially its context and effects, remains limited in many countries, including Norway. A few studies have done more scoping surveys on psychedelics and MDMA together. Cuttler et al. (5) reported from a survey covering both and found that mean distress ratings among users were low. Lake and Lucas (6) found that personal growth was the most common motivation in English-speaking regions. Rojas-Hernández et al. (7) reported that MDMA led to the most adverse reactions in Spanish- speaking countries, compared to other psychedelic drugs. Whelan et al. (8) addressed harm and harm reduction among MDMA users in an area of New Zealand, finding that 14 per cent reported harm from MDMA use, and about 7 per cent of the sample were described as dependent.

Clinical use of MDMA on the other hand, or 3,4-Methylenedioxymethamphetamine by its chemical formulation, has gained renewed interest after showing promising effects in the treatment of posttraumatic stress disorder (9–11). The Food and Drug Administration in the US (FDA) designated MDMA as a Breakthrough Therapy in 2017 (12), a status aimed at expediting the development and review of a drug.

MDMA is a synthetic amphetamine derivative with stimulant and hallucinogenic properties (11). MDMA’s subjective effects are attributed to its impact on monoamine neurotransmission. Specifically, MDMA triggers the release of serotonin, dopamine, and norepinephrine, while also inhibiting their reuptake. This surge in monoamine activity is responsible for the characteristic feelings of euphoria, empathy, and sociability associated with MDMA use. Although toxicity is regarded as relatively low, prolonged and high-dose use has been associated with tolerance, depression, and cognitive deficits (13).

Strict guidelines are therefore implemented for clinical use, but even more focus is directed at the interplay between the physical effects of MDMA, the setting, and its relational aspects (14).

MDMA can be considered a mood enhancer. Since mood is susceptible to many influences, contextual factors, motivation, and intention may also play a role in the experience with the substance (15). Factors related to set and setting are described as relevant to the outcome of MDMA use, both clinically and recreationally (16, 17). A study of MDMA use in couples describes the couples’ reflection on the framework and context for the experience in terms of communication, intimate bonding, and relationship focus (18), highlighting the decisive role of set and setting in determining the quality of their shared experiences. Recreational use where participants report increased insight as the main motivation for use indicates more favorable outcomes than more casual use (2). Risk perception may be diminished under MDMA influence, but this may be due to its empathogenic effects (19). The “come-down” effect, where a person might experience two to five days of depressed mood, may also be a set and setting effect (20), although this study has been criticized (21) and results are generally mixed (22–24). Even associations with the substance, such as taking “ecstasy” in contrast to “molly”, can affect the experience, even if the active substance in the comparative drug is possibly the same (25). Thus, motivation, environment, aftercare, and presence and support from others can play a role both in the active phase and in the MDMA experience more broadly. Examining set and setting is therefore crucial if we want to understand what might separate beneficial from adverse effects of MDMA. It is also crucial to understand whether set and setting as applied in clinical studies is determining the recreational MDMA experience. A less strict framing of the experience as seen in much of recreational use may also lead to positive outcomes and still shield against negative outcomes. The latter would imply that clinical setups can be somewhat simplified, and inclusion criteria may also be somewhat relaxed.

Several studies report on positive health effects of recreational MDMA use. During the COVID-19 pandemic, users in the UK who were initially more troubled by mental health issues reported improvements after psychedelics and/or MDMA use (26). A study from across Western countries reported long-term socio-emotional benefits for subgroups setting self-insight as an important goal (2). At the same time, users also report adverse effects from MDMA use, and more so than from other substances (7). Is this indicative of how set and setting shape the experience? Polydrug use is also a complicating factor in this picture, a high proportion of users report simultaneous use of alcohol and other drugs (7, 27).

To address the knowledge gap in MDMA-specific contexts and effects, we conducted a large-scale online survey to investigate the patterns and characteristics of recreational MDMA use in a self-selected sample. Building on our previous research on classic psychedelic substances in Norway (28), this study describes key factors shaping MDMA’s effects and outcomes. We examine patterns of use, set and setting, social support, intentions, preparation, co-use of other substances, and post-use integration. We also investigate the distribution of reported psychological and emotional experiences, adverse events, addiction, personality factors, psychiatric and neurodevelopmental comorbidities, and changes in concurrent drug use and suicidality. These insights are essential for understanding why people use MDMA, its potential psychological, emotional, social, and health-related benefits, as well as its adverse outcomes – key knowledge for shaping effective public health policies and harm reduction strategies.

## Methods

### Recruitment and participant characteristics

We conducted an anonymous online survey comprising of 150 items using Nettskjema, a survey solution developed and hosted by the University of Oslo. The participants were asked about a memorable experience after taking MDMA. Psychedelic experiences from classical psychedelics were surveyed in a previous study by the same research group and were therefore not targeted in this study. Participants were recruited via the web pages and social media groups of the Norwegian Association for Psychedelic Science, Rusopplysningen.no – an information service about commonly used drugs – and numerous Facebook-pages for people with an interest in psychedelics. Participation in the study was voluntary and anonymous, and participants did not receive any form of compensation. They were informed that even after they started the survey, they could cancel at any time, and if they did, none of their answers would be stored. In the final question, participants had to confirm whether their data could be used in the study.

Participants completed the survey from March 6^th^ to August 28^th^, 2024. Inclusion criteria were 1) being 18 years or older, 2) fluent in Norwegian (reading, writing, and speaking), and 3) having had a memorable experience with MDMA within the past five years. Responding no to any of these questions led to exclusion from the data set. Participants were asked to report their gender, age, education level, annual income group, and marital status. Due to concerns about data sensitivity and participant identification, we did not ask for other demographic data. They also reported the frequency of MDMA use across their lifetime, and within the past year.

### Characteristics of the most memorable MDMA experience

Participants responded to the following questions regarding the most memorable MDMA experience: to what extent they remembered the experience, the setting in which MDMA was taken, whether they had any support during the experience or paid someone to guide them through the experience, motivation to take MDMA (therapeutic or other), collaboration with therapist, guide or trip-sitter if any, dosage, preparation, degree of clear intention, wish for change, co-administration of alcohol or other substances, and integration after the experience. Participants were asked to which degree they agreed that MDMA should be used therapeutically.

### Effects of MDMA use

Participants were asked about any increased understanding of the significance of past life events during the experience, to what extent they uncovered any associations between current and past interpersonal relationships during the experience, and to what extent they discovered any new or forgotten ways of dealing with difficulties and challenges during the experience.

We also asked the participants about the occurrence of persisting adverse reactions, and the severity and duration of such reactions after the MDMA experience, and if they sought professional help to handle adverse reactions. We also asked about craving symptoms and failed attempts to resist new intake of the substance.

We asked the participants to compare their most memorable experience to other meaningful experiences in life, i.e., how meaningful, how spiritual, how psychologically challenging and insightful the experience was. Furthermore, we asked about subjectively perceived persisting changes in personal well-being/life-satisfaction, purpose and meaning of life, social relationships, mood, behavior, spirituality, as well as attitudes about life, nature, and death.

### Standardized questionnaires

We also included four standardized questionnaires in the survey: the Psychological Insight Scale (PIS; 29), the Emotional Breakthrough Inventory (EBI; 30a), the Challenging Experience Questionnaire (CEQ; 31a), and the Level of Personality Functioning Scale – Brief Form (LPFS-BF; 32). The first three instruments have been translated and examined for psychometric properties in relation to the study on psychedelics (28), and publications of these examinations are currently underway (33). The Norwegian version of LPFS-BF has been translated and validated by other researchers (34).

PIS is a seven 7-item questionnaire assessing change in insight following a psychedelic experience, and behavioral change (29). It is scored on a scale with the anchors 0 to 100, where 0 represents “no more than usual” and 100 represents “more than usual”. An example item is “I have learned important new ways of thinking about my ‘self’ and my problems”. Increased PIS score represents increased insight.

EBI is a 6-item questionnaire that was developed to map emotional breakthrough following a psychedelic experience (30). The EBI consists of six statements scored on a scale from 0 (“no, not more than usual”) to 100 (“yes, entirely or completely”). An example of an item from the questionnaire is “I faced emotionally difficult feelings that I usually push aside.” Higher EBI score represents a higher level of emotional breakthrough.

CEQ is a 26-item self-report questionnaire measuring perceived challenging aspects of a psychedelic experience (31). Participants were asked to rate their most memorable experience with a classic psychedelic substance on a five-point Likert scale ranging from 0 (“none: not at all”) to 5 (“extreme”). The CEQ is divided into seven dimensions: 1) Isolation, 2) Grief, 3) Physical distress, 4) Fear, 5) Insanity, 6) Paranoia, and 7) Death. An example of an item from the questionnaire is “Isolation and loneliness.” Higher CEQ scores represent higher degrees of perceived challenges.

LPFS-BF is a 12-item self-report questionnaire designed to assess an individual’s overall level of personality functioning (32, 35). The items are rated on a scale from 0 (“not at all”) to 3 (“very much”). A sample item from the LPFS is “I often don’t know who I really am”. Higher LPFS-BF scores represent higher levels of functional impairment. For each of the 12 items, participants also indicated whether the aspect in question had changed following the MDMA experience, on a scale from -5 (“worse”) to 5 (“better”) with 0 as a neutral mid-point.

### Mental disorders and substance use disorders

We asked the participants if they had been diagnosed with or suspected that they would meet the criteria for the following mental disorders (“psykiske lidelser” in Norwegian), and whether the condition was diagnosed by a professional: social anxiety, panic disorder, OCD, depression, bipolar disorder, psychosis/schizophrenia, eating disorder, PTSD, attention deficit hyperactivity disorder (ADHD), autism spectrum disorder (ASD), and suicidality. Furthermore, we asked about substance use disorders, including nicotine dependence (daily smoking), and abuse of or addiction to alcohol or other substances. The latter included illegal drugs other than MDMA, and addictive prescription drugs. We asked about concurrent use of the following drugs together with the memorable MDMA experience: dissociatives (ketamine, PCP, etc.), cannabis (hashish, marihuana, THC), stimulants (amphetamine, methamphetamine, Ritalin), cocaine, alcohol, psychedelics (LSD, Psilocybin/magic mushroom, 2-CB, Ayahuasca, DMT [N,N-dimethyltryptamine], 5-MeO-DMT [5-methoxy-N,N-dimethyltryptamine], Peyote/mescaline), opiates (morphine, heroin, methadone, OxyContin, codeine, opium), inhalants (glue, ethyl chloride, nitrous oxide, amyl nitrate or butyl nitrate), anxiolytics (benzodiazepines, Valium, Vival, Stesolid, Xanor, Ativan, Rivotril, barbiturates, GHB, Rohypnol), and other.

### Ethical considerations and data protection

In the consent form, it was stated that the survey should not be interpreted as an endorsement of MDMA or an encouragement for its use. We did not collect identifiable information such as name, email address or IP address. A preliminary request was submitted to the regional committee for medical and health research ethics (REK, reference number 212268). Because we only collected anonymous data, the committee deemed the study exempt from full evaluation, and we were allowed to proceed with the data collection. The study was subsequently approved by the Internal ethics committee at the Department of Psychology, University of Oslo (reference number 18183844). We used a secure web application for data storage (Services for Sensitive Data; TSD), which meets requirements for the processing and storage of sensitive research data.

### Participant and public involvement

Prior to the data collection, the survey was reviewed by a focus group of five volunteers recruited from the Norwegian Association for Psychedelic Science as well as a designated user representative at Østfold Hospital Trust. Their task was to flag problematic, incomprehensible or ambiguous questions. As a result of feedback from the focus group, we changed the wording of some questions, but no questions were added or removed.

### Statistical analysis

We present descriptive statistics for demographics, previous use of MDMA, lifetime history of self-reported mental disorders and substance use disorders, characteristics of the most memorable MDMA experience, and persisting adverse reactions and benefits from the experience. We used chi-square-tests and correlations (Pearson’s *r*, Cramer’s V) to investigate gender and age differences. Analyses were performed using IBM SPSS v30.0.

## Results

### Respondent characteristics

**Figure 1:**
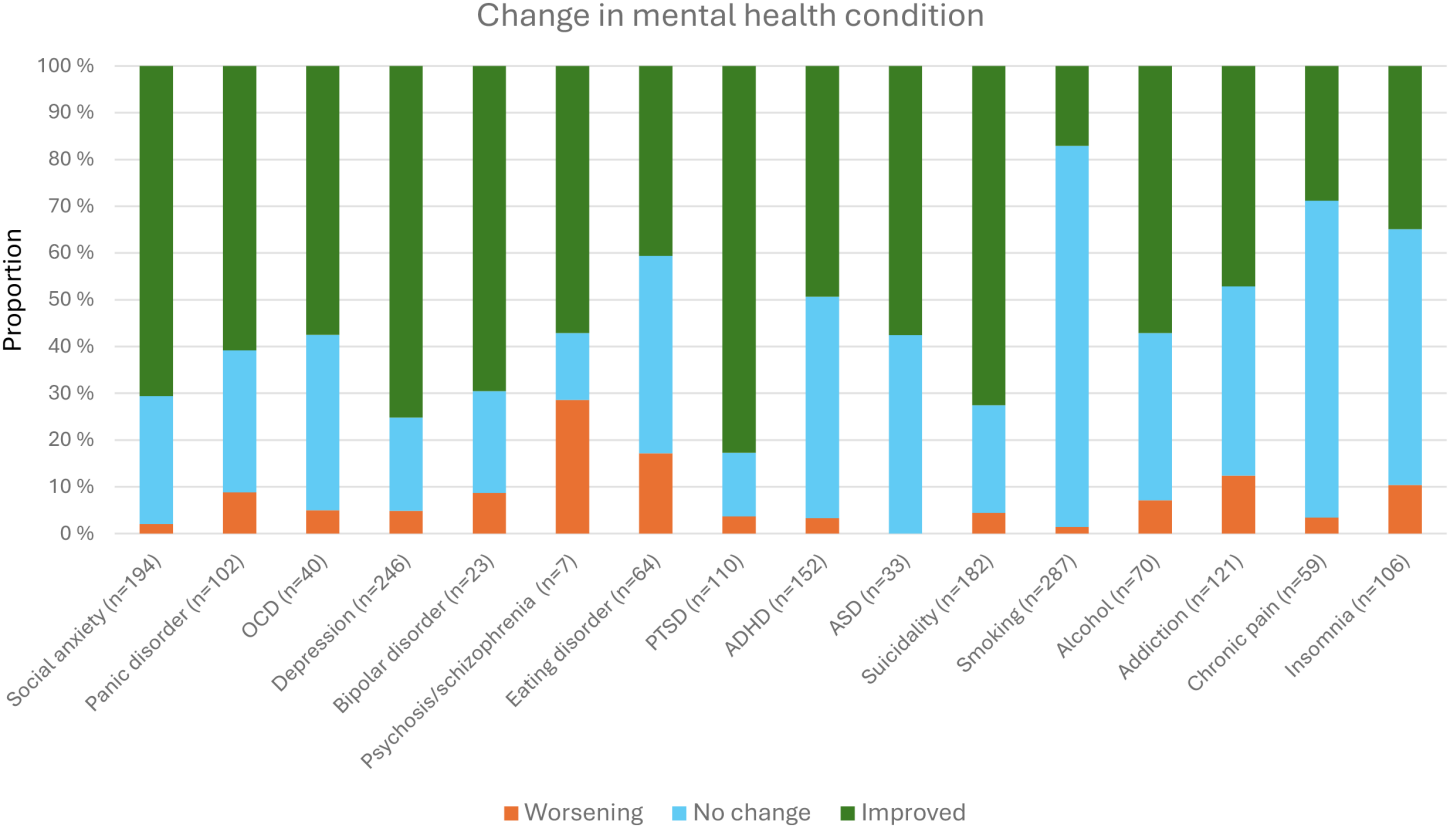
Reasons for excluding 71 participants from the data analysis (numbers overlap).

Of 654 participants who completed the survey, 608 were deemed eligible for the data analysis, 366 of which reported MDMA without concomitant use of other substances. See **Figure 1** for details of participant exclusion and selection of subsamples. Of the 608 included in the total sample, 234 were female (38.5%), 368 male (60.5%) and six reported as non-binary (1%, see **Table 1** for demographics). Most participants were 45 years old or younger (89%), 66.3% were under 36, all were under 65 years of age. On income, 16.3% reported below NOK 300,000 in yearly income, which is well below poverty levels, whereas 11.7% reported above NOK 1,500,000, which is more than double the average income in Norway, which is NOK 712,440 (36). Just over half of the participants reported some degree of higher education, just less than half reported their marital status as “single”.

**Table 1:** Demographics.

Reponse option	n (%)
Gender	
Female	234 (38.5%)
Male	368 (60.5%)
Non-binary	6 (1.0%)
Age group	
18–25 years	175 (28.8%)
26–35 years	228 (37.5%)
36–45 years	138 (22.7%)
46–55 years	52 (8.6%)
56–65 years	15 (2.5%)
66+ years	0 (0.0%)
Educational level	
Not completed primary school^1^	3 (0.5%)
Completed primary school	52 (8.6%)
Completed upper secondary school/high-school^2^	184 (30.3%)
Completed vocational school^2^	56 (9.2%)
Completed higher education (university/college) up to 4 years	157 (25.8%)
Completed higher education (university/college) more than 4 years	156 (25.7%)
Annual income (NOK)	
Less than 300 000	99 (16.3%)
300000-399 000	48 (7.9%)
400000-499 000	49 (8.1%)
500000-749 000	146 (24.0%)
750000-999 999	87 (14.3%)
1000000-1249 999	61 (10.0%)
1250000-1499 999	47 (7.7%)
1500000 or more	71 (11.7%)
Relationship status	
Married	86 (14.1%)
Cohabitant	184 (30.3%)
Divorced or separated	33 (5.4%)
Widower/widow	2 (0.3%)
Single	303 (49.8%)
Employment status	
Working full time	366 (60.2%)
Working part-time	54 (8.9%)
Unemployed	7 (1.2%)
Disability benefit	58 (9.5%)
Pensioner	1 (0.2%)
Pupil/student	122 (20.1%)

*N* = 608. ^1^10 years, ^2^3 years

### Previous use and other substances

**Figure 2:**
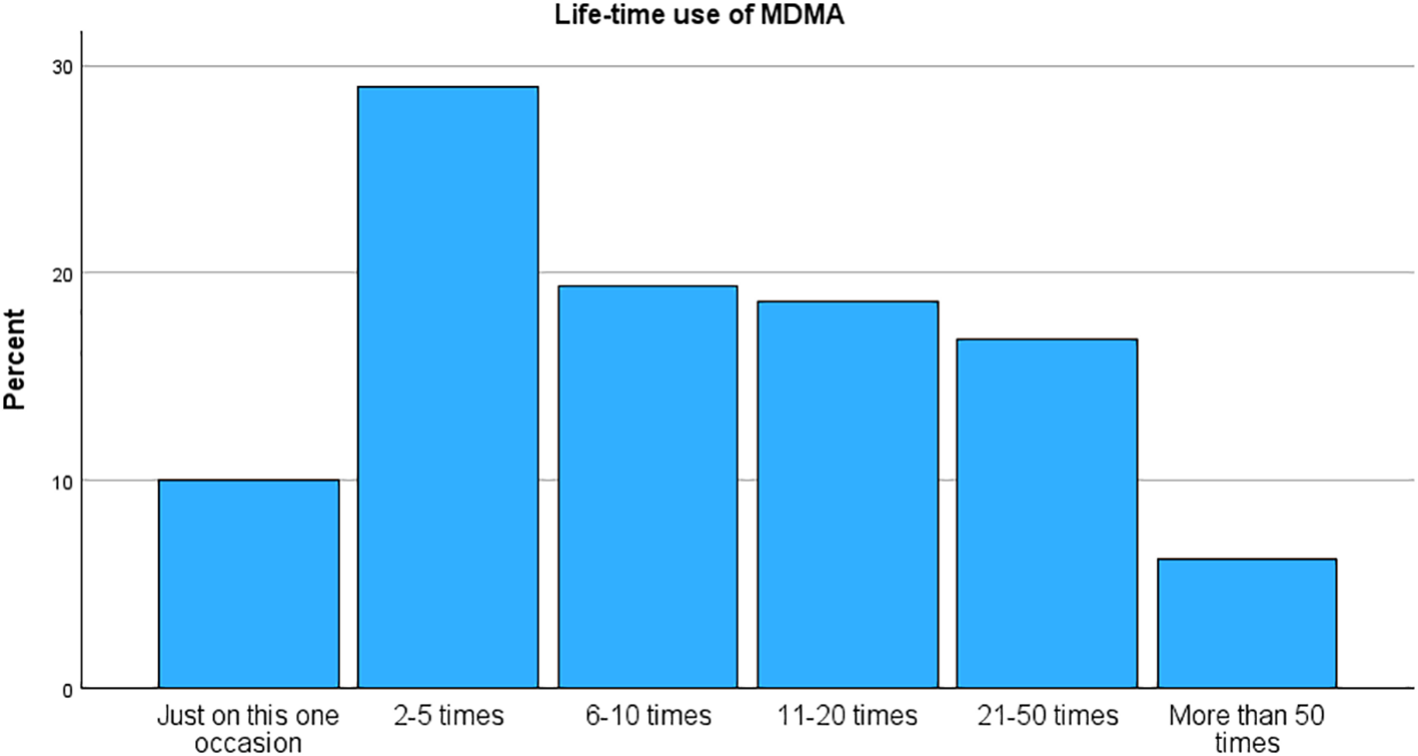
Life-time use of MDMA.

**Table 2** shows previous use of MDMA and motivation, and **Table 3** shows the use of other substances. A small proportion of the sample – 10.0% – reported only one experience with MDMA, and 31.4% reported no use last year. Of the remaining, 48.3% reported MDMA use one to ten times in the past year, 41.7% reported more than ten times (see **Figure 2**). Five participants (0.8%) reported using MDMA more than 50 times over the previous year. We found no connection between frequency of use and gender (*χ^2^* = 15,600, *df* = 10, *p* = .112, Cramer’s V = .11), but there was a small positive correlation with age (*r* = .14, *p* <.001).

**Table 2:** Previous use.

Reponse option	n (%)
Life-time use of MDMA	
Just on this one occasion	61 (10.0%)
2-5	176 (28.9%)
6-10	118 (19.4%)
11-20	113 (18.6%)
21-50	102 (16.8%)
More than 50	38 (6.3%)
Past year use of MDMA	
No use last year	191 (31.4%)
Only this one time	91 (10.0%)
2-5	250 (41.1%)
6-10	51 (8.4%)
11-20	17 (2.8%)
21-50	3 (0.5%)
More than 50	5 (0.8%)
Motivation for using more than once	
Did not achieve desired effect first time	18 (3.0%)
Wanted the same experience again	492 (80.9%)
Not a conscious choice	42 (6.9%)
Only once	56 (9.2%)

*N* = 608.

**Table 3:** Substance intake. .

Reponse option	n (%)
Concurrent substance intake (yes/uncertain^1^)	
Only MDMA	366 (60.2%)
MDMA and other substance	242 (39.8%)
Dissociatives (ketamine, PCP, etc.)	31 (12.8%)
Cannabis (hashish, marihuana, THC)	94 (38.8%)
Stimulants (amphetamine, methamphetamine, Ritalin)	43 (17.8%)
Cocaine	42 (17.4%)
Alcohol	145 (59.9.%)
Psychedelics (LSD, Psilocybin/magic mushroom, 2-CB, Ayahuasca, DMT (N,N-dimethyltryptamine), 5-MeO-DMT (5-methoxy-N,N-dimethyltryptamine), Peyote/mescaline)	44 (18.2%)
Opiates (morphine, heroin, methadone, OxyContin, codeine, opium)	5 (2.1%)
Inhalants (glue, ethyl chloride, nitrous oxide, amyl nitrate or butyl nitrate)	1 (0.4%)
Anxiolytics (benzodiazepines, Valium, Vival, Stesolid, Xanor, Ativan, Rivotril, barbiturates, GHB, Rohypnol)	15 (6.2%)
Other^2^	0
Perceived impact of dose	
Low	27 (4.4%)
Moderate	388 (63.8%)
High	193 (31.7%)
How well the experience remains in memory	
Not very	5 (0.8%)
Somewhat	54 (8.9%)
Quite well	255 (41.9%)
Very well	294 (48.4%)

*N* = 608. ^1^16 participants across categories. ^2^Four “Other” entries were recoded into a specific category based on text input.

Over 80% reported that they wanted the same experience again as motivation for repeated use. Most of the participants used only MDMA (60.2%), whereas alcohol was the most frequent other substance together with MDMA (59.9% of those with mixed use). Simultaneous cannabis use was also frequent (38.8%), whereas other substances were used more scarcely (less than 18.2%). No gender differences were observed in concurrent drug use neither overall nor for cannabis or alcohol (*p* = .110 to.300). For age, there was a sharp drop-off over 45 years where almost no one reported taking other drugs besides MDMA (*χ^2^* = 16,280, *df* = 8, *p* = .039, Cramer’s V = .16). When asked about their perception of the dose based on the effects of MDMA, most respondents (95.5%) described it as moderate or high. Over 90% reported that they remembered the experience quite or very well.

### Set and setting

**Figure 3:**
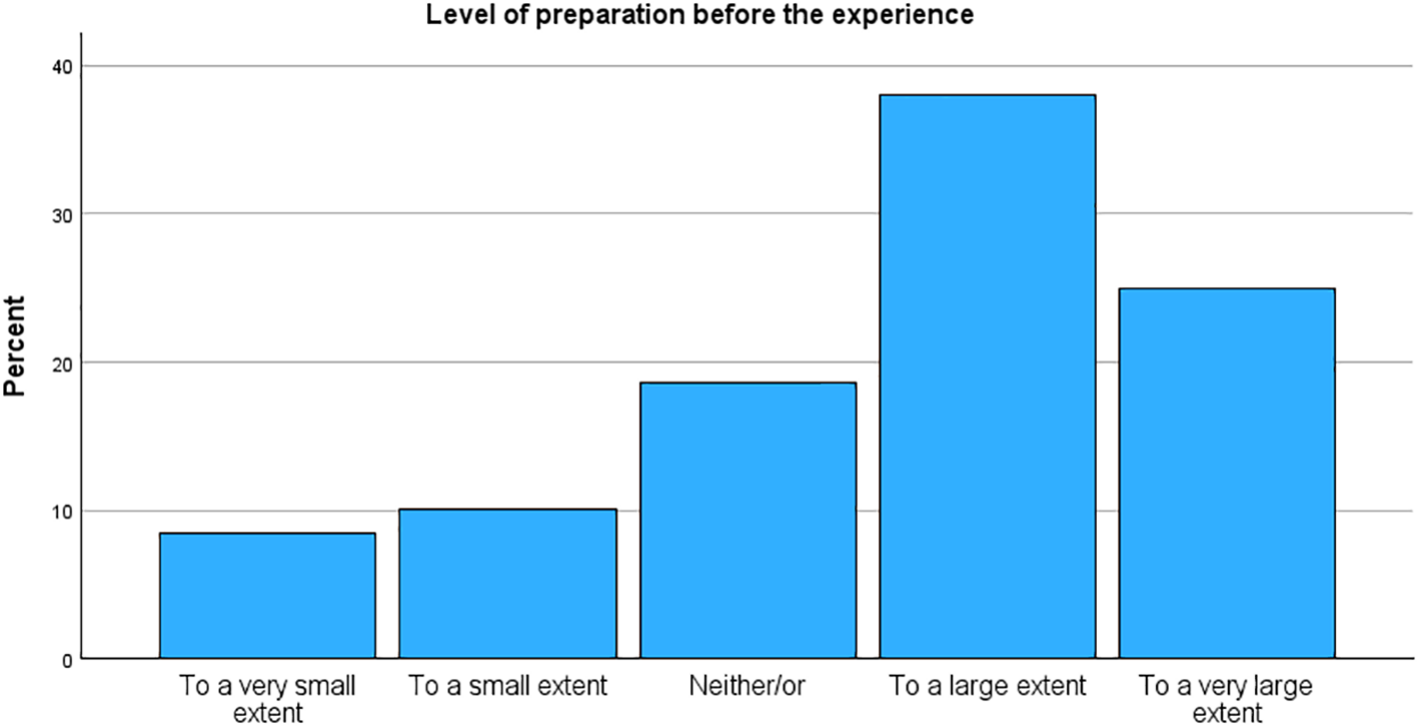
Level of preparation before the experience.

**Figure 4:**
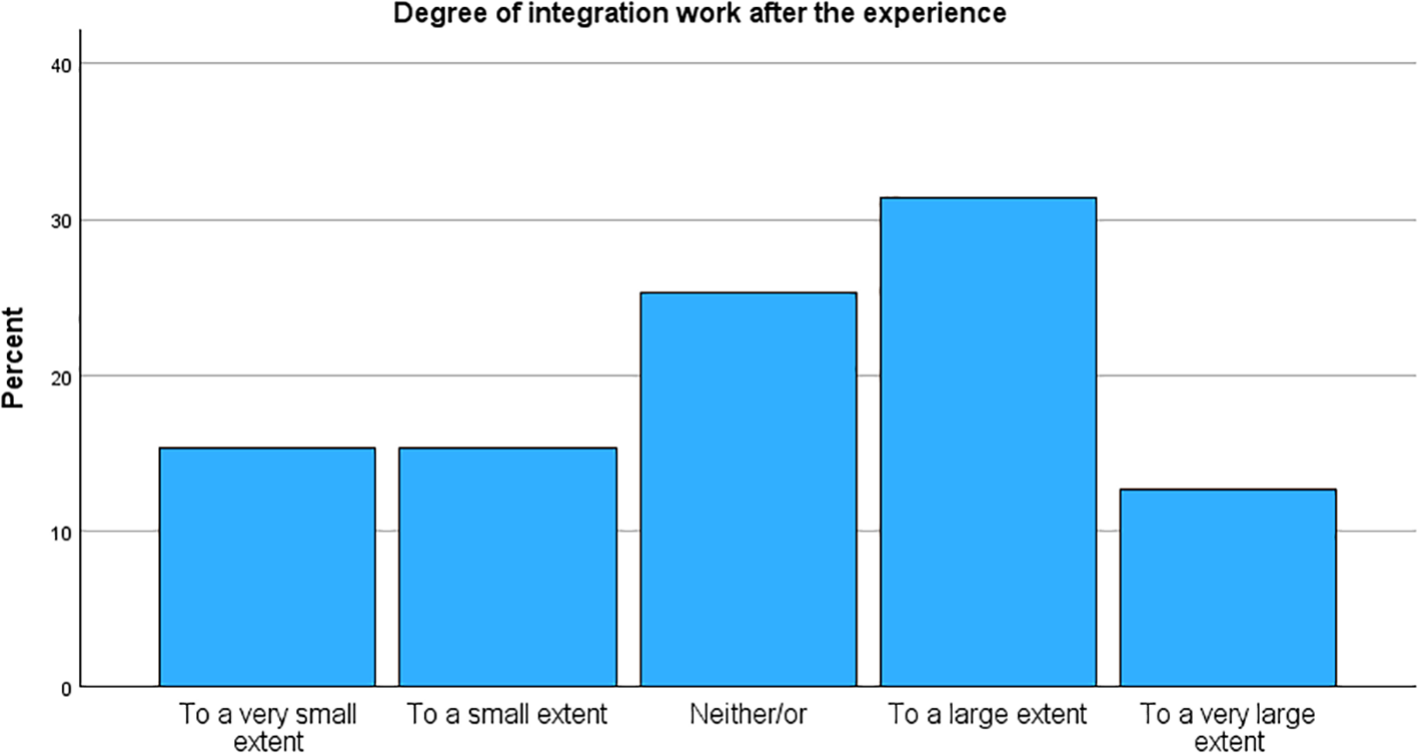
Degree of integration work after the experience.

**Table 4** details the reported set and setting of the experience, and **Table 5** reports on experiences and attitudes. Most participants used MDMA at someone else’s home (34.4%) or at home (28.3%), a total of 62.7% in a private setting. 48.8% received guidance or support, and only 5.8% were alone. Nine participants reported paying a guide, which could be indicative of a so-called “underground clinic”, meaning someone offering guidance and/or therapy as an illicit service. The vast majority regarded support or guiding as somewhat or very constructive (78.9%). A majority of 65.6% reported taking MDMA as a purely recreational activity, whereas 22.9% reported a therapeutic setting. Most participants prepared for the occasion to a large or very large extent (63.0%), and a significant portion had a clear intention to a large or very large extent (54.3%, see **Figure 3**). The sample was mixed on their report of desire for change, with the largest portion reporting neither/or (35.7%). Still, a large portion also reported integration work as part of the experience to a large or very large extent (44.1%, see **Figure 4**). A clear majority agree or strongly agree that MDMA should be used therapeutically (84.7%, *M* = 4.40, *SD* = 0.89). Participants reported a clear increase in their understanding of life events (*M* = 3.67, *SD* = 1.57), in their discovery of relational patterns (*M* = 4.00, *SD* = 1.50), and new ways of coping (*M* = 3.57, *SD* = 1.58). Participants were minimally concerned about the illegal status of the drug (*M* = 2.70, *SD* = 1.43), and merely 20 participants (3.2%) reported that their MDMA use had resulted in encounters with the police.

**Table 4:** Set and setting.

Reponse option	n (%)
Setting in connection with the experience	
At home	172 (28.3%)
At somebody else’s home	209 (34.4%)
Outdoors, in nature	39 (6.4%)
Outdoors, urban environment	40 (6.6%)
Church/other religious or ceremonial/spiritual setting	4 (0.7%)
At a festival	68 (11.2%)
Therapeutic clinic or with a guide/trip sitter	12 (2.0%)
Other	64 (10.5%)
Support during the experience	
Alone	35 (5.8%)
Not alone, but did not receive guidance or support	276 (45.4%)
Received guidance or support	297 (48.8%)
With a paid guide	9 (1.5%)
Motivation for taking MDMA	
Recreational, for fun or out of curiosity	399 (65.6%)
Therapeutic: desire for gained insight, reduction of symptoms, increased quality of life or processing of difficult memories or feelings	139 (22.9%)
Wish for a spiritual or religious experience	20 (3.3%)
Escape or distraction from discomfort, challenges in life, or boredom	50 (8.2%)
Collaboration with a therapist, guide or trip-sitter	90 (14.8%)
Very unconstructive	1 (1.1% of 90)
Neither/or	18 (20.0% of 90)
Somewhat constructive	18 (20.0% of 90)
Very constructive	53 (58.9% of 90)
Not applicable (did not take the psychedelic drug with a therapist)	518 (85.2%)
Level of preparation before the experience	
To a very small extent	51 (8.4%)
To a small extent	61 (10.0%)
Neither/or	113 (18.6%)
To a large extent	231 (38.0%)
To a very large extent	152 (25.0%)
Level of intention in connection with the experience	
To a very small extent	59 (9.7%)
To a small extent	77 (12.7%)
Neither/or	142 (23.4%)
To a large extent	226 (37.2%)
To a very large extent	104 (17.1%)
Desire for change in connection with the experience	
To a very small extent	125 (20.6%)
To a small extent	126 (20.7%)
Neither/or	217 (35.7%)
To a large extent	96 (15.8%)
To a very large extent	44 (7.2%)

*N* = 608.

**Table 5:** Experiences and attitudes following MDMA use.

Response option	n (%) or M (SD)
Degree of integration work after the experience	
To a very small extent	93 (15.3%)
To a small extent	93 (15.3%)
Neither/or	154 (25.3%)
To a large extent	191 (31.4%)
To a very large extent	77 (12.7%)
Agreement whether MDMA should be used therapeutically (1-5)	4.40 (0.89)
Strongly disagree	6 (1.0%)
Disagree	22 (3.5%)
Neither/or	67 (10.8%)
Agree	148 (23.8%)
Strongly agree	378 (60.9%)
Degree of increased understanding of life events (1-5)	3.67 (1.57)
Not at all	76 (12.2%)
Minimal	93 (15.0%)
Somewhat	84 (13.5%)
Moderately	150 (24.2%)
Strongly	140 (22.5%)
Extremely	78 (12.6%)
Degree of discovery of relational patterns (1-5)	4.00 (1.50)
Not at all	56 (9.0%)
Minimal	63 (10.1%)
Somewhat	83 (13.4%)
Moderately	130 (20.9%)
Strongly	200 (32.2%)
Extremely	89 (14.3%)
Degree of new ways of coping (1-5)	3.57 (1.58)
Not at all	92 (14.8%)
Minimal	82 (13.2%)
Somewhat	103 (16.6%)
Moderately	128 (20.6%)
Strongly	155 (25.0%)
Extremely	61 (9.8%)
Stated effect of illegal status (1-5)	2.70 (1.43)
Not at all	164 (36.4%)
Minimal	144 (23.2%)
Somewhat	125 (20.1%)
Moderately	109 (17.6%)
Strongly	61 (9.8%)
Extremely	18 (2.9%)
Difficulties with the police in connection with the experience	
Yes	20 (3.2%)
No	601 (96.8%)

*N* = 608.

**Table 6** shows a comparison to other experiences and persisting changes. The experience was considered, from 1-8, highly meaningful (*M* = 5.11, *SD* = 1.66), spiritual (*M* = 4.04, *SD* = 2.33), and insightful (*M* = 4.69, *SD* = 2.00), but not very challenging (*M* = 2.56, *SD* = 1.80). The most prominent persistent changes were attitudes towards death (*M* = 3.64, *SD* = 1.03), spirituality (*M* = 3.29, *SD* = 1.01), and attitudes towards nature (*M* = 3.09, *SD* = 1.13). Additionally, about one fifth of the participants rated the experience as among the top five or the single most personally meaningful, spiritual, or psychologically insightful experience (range 19-24%).

**Table 6:** Comparison to other life experiences and change attributed to the experience.

Response option	M (SD)
Comparison to other lifetime experiences (1–8, least to most significant)	
Personally meaningful	5.11 (1.66)
Spiritual	4.04 (2.33)
Psychologically insightful	4.69 (2.00)
Psychologically challenging	2.56 (1.80)
Degree of persisting changes after the experience (1-7, least to most change)	
Life satisfaction and general well-being	2.38 (1.25)
Purpose in life	2.65 (1.20)
Meaning of life	2.76 (1.17)
Social relationships	2.49 (1.32)
Attitudes towards life	2.57 (1.14)
Attitudes towards nature	3.09 (1.13)
Mood	2.96 (1.24)
Behavior	3.05 (1.16)
Spirituality	3.29 (1.01)
Attitudes towards death	3.46 (1.03)

*N* = 608.

### Standardized questionnaires

**Table 7** shows the results from the four standardized questionnaires: PSI, EBI, CBQ and LPFS-BF. Respondents reported a high degree of psychological insight following the experience (PIS *M* = 51.29, *SD* = 30.24) as well as a high degree of emotional breakthrough (EBQ *M* = 49.83, *SD* = 28.69). At the same time, they reported a fairly low level of challenging experiences (CEQ *M* = 1.56, *SD* = 0.67). Physical distress was the most common challenge (*M* = 2.12, *SD* = 1.00), followed by grief (*M* = 1.59, *SD* = 0.84) and fear (*M* = 1.51, *SD* = 0.89). Participants reported positive personality changes on average, but the individual variance was high (*M* = 0.73, *SD* = 1.06).

**Table 7:** Scores on standardized questionnaires.

Questionnaire scores	M (SD)
Psychological Insight Scale (PIS, 0-100)	51.29 (30.24)
Emotional Breakthrough Inventory (EBI, 0-100)	49.83 (28.69)
Challenging Experiences Questionnaire (CEQ, 0-5)	1.56 (0.67)
CEQ Isolation (3 items)	1.46 (0.86)
CEQ Grief (6 items)	1.59 (0.84)
CEQ Physical distress (5 items)	2.12 (1.00)
CEQ Fear (5 items)	1.51 (0.89)
CEQ Insanity (3 items)	1.29 (0.73)
CEQ Paranoia (2 items)	1.18 (0.62)
CEQ Death (2 items)	1.14 (0.64)
Level of Personality Functioning Scale – Brief Form (LPFS-BF, 1-4)	1.75 (0.51)
Perceived change^1^	0.73 (1.06)
LPFS Self-functioning (6 items)	1.93 (0.66)
Perceived change^1^	0.86 (1.29)
LPFS Interpersonal functioning (6 items)	1.56 (0.49)
Perceived change^1^	0.59 (1.00)

*N* = 608. ^1^Rating of perceived change on each item after MDMA experience, rated from -5 (large negative change) to 5 (large positive change)

### Perceived change of mental disorders and substance use disorders

**Figure 5:**
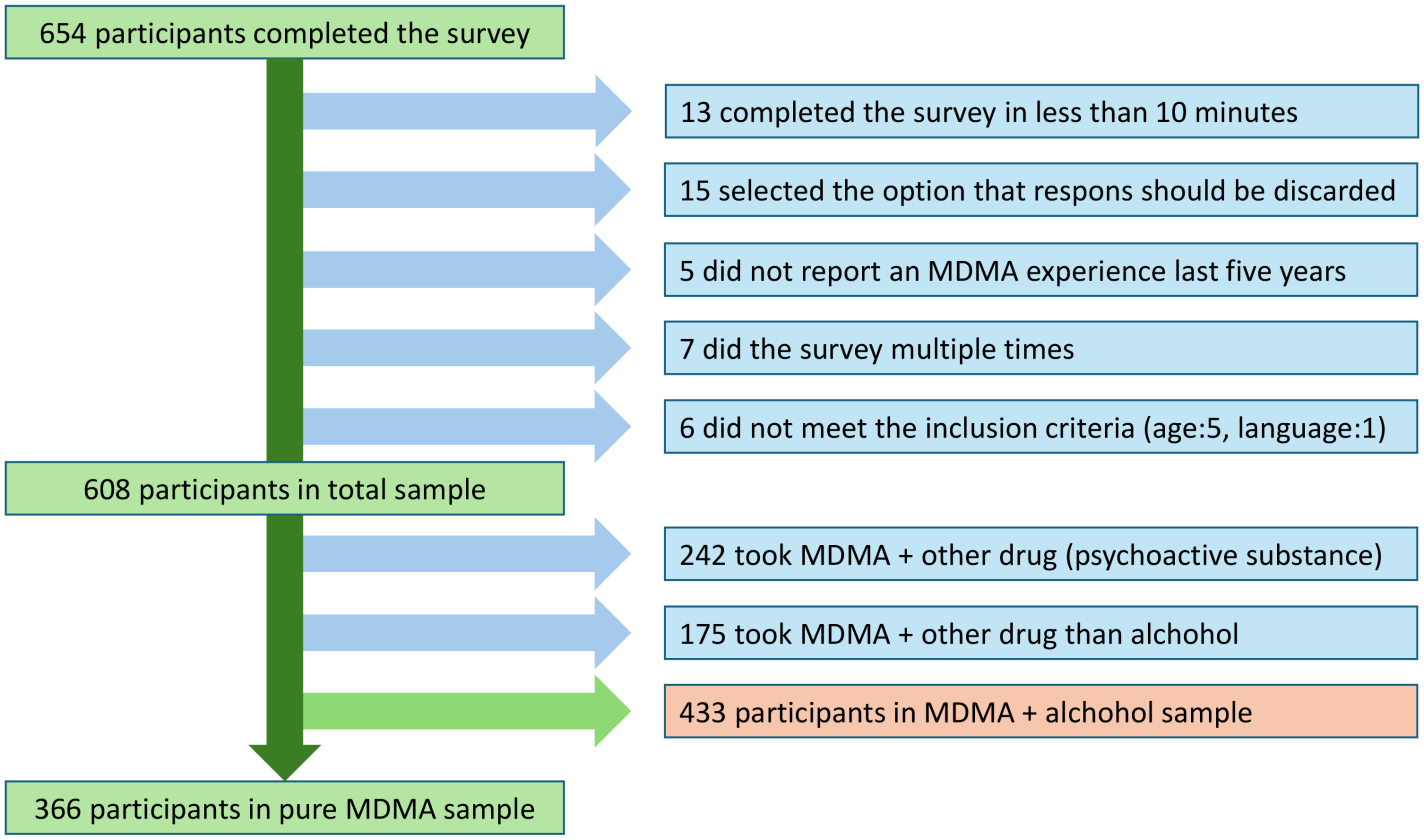
Number of diagnoses.

As shown in **Tables 8** and **9**, the most prevalent self-perceived or clinician-assessed diagnoses were smoking (47.5%), depression (40.5%), social anxiety (31.9%), suicidality (29.9%), and ADHD (25.4%). The mean number of diagnoses was 2.96 (SD 2.68). As displayed in **Figure 5**, most of the participants (499, 82.1%) reported at least one diagnosis, whereas only 109 participants (17.9%) reported no mental disorders or substance use disorders.

**Table 8:** History of mental and substance use disorders. .

Self-reported mental disorder	Frequency (n/%)	Degree of change (M/SD)
Social anxiety	194 (31.9%)	2.71 (1.19)
Panic disorder	102 (16.8%)	2.97 (1.34)
OCD (obsessive-compulsive disorder)	40 (6.6%)	3.23 (1.19)
Depression	246 (40.5%)	2.59 (1.36)
Bipolar disorder	23 (3.8%)	2.88 (1.64)
Psychosis/schizophrenia	7 (1.2%)	4.00 (1.85)
Eating disorder	64 (10.5%)	3.62 (1.64)
PTSD (post-traumatic stress disorder)	110 (18.1%)	2.27 (1.29)
ADHD (attention-deficit/hyperactive disorder)	156 (25.7%)	3.28 (1.02)
ASD (autism spectrum disorder)	33 (5.4%)	2.86 (1.40)
Suicidality	182 (29.9%)	2.58 (1.40)
Nicotine dependency	287 (47.5%)	3.72 (0.83)
Alcohol abuse/addiction	70 (11.5%)	2.89 (1.55)
Other abuse/addiction	121 (19.9%)	3.36 (1.40)
Chronic pain	59 (9.7%)	3.57 (1.01)
Insomnia	106 (17.4%)	3.57 (1.20)
Registered diagnose*	313 (51.5%)	

*N* = 608. *Participant reporting that the diagnosis is given by a psychologist or a psychiatrist.

**Table 9:** Crosstabs of diagnostic status.

	*Self-reported diagnosis (n/%)*	*Registered diagnosis (n/%)^1^*
No diagnosis	109 (17.9%)	
At least one diagnosis	499 (82.1%)	294 (48.4%)
Mental disorder^2^	399 (65.6%)	263 (43.3%)
Other disorder^3^	100 (16.4%)	31 (5.1%)

*N* = 608. ^1^Participant reporting that the diagnosis is given by a psychologist or a psychiatrist. ^2^Mental disorder is defined as social anxiety, panic disorder, OCD, depression, bipolar disorder, psychosis/schizophrenia, eating disorder, PTSD, suicidality, and alcohol/substance abuse. ^3^Other disorder defined as ADHD, ASD, nicotine dependency, chronic pain, and insomnia.

**Figure 6:**
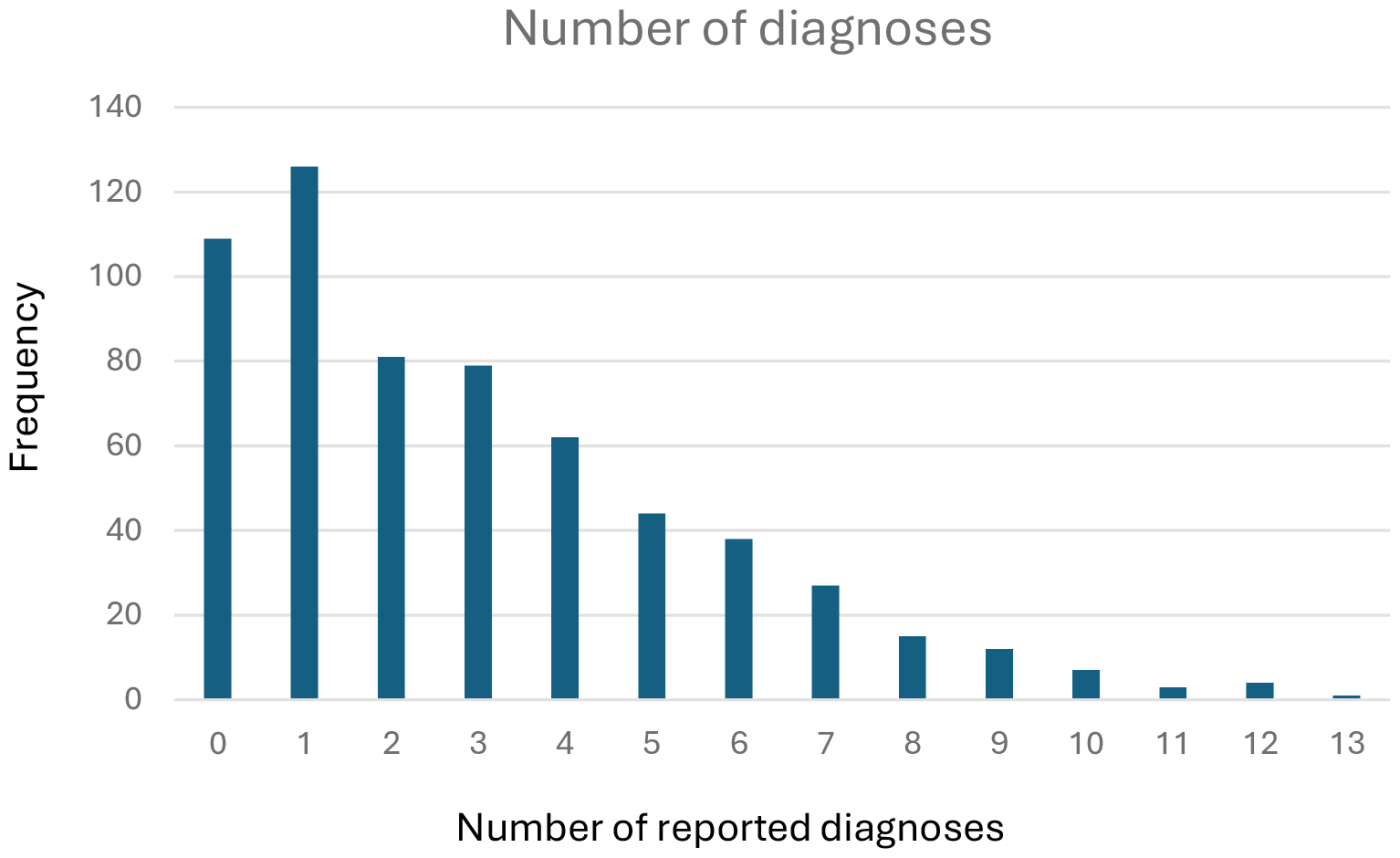
Proportion of perceived change in mental health condition.

**Figure 6** shows the proportion of reported change for each condition, grouped into worsening (small, medium or large unwanted change), no change, or improved (small, medium or large desired change). The largest negative change was reported for psychosis of 29%, although the numbers were small (n=7). Eating disorder had a 17% negative change, whereas addiction had 12%, the remaining were all at 10% or less. The largest proportion of no change occurred for smoking (82%), chronic pain (68%), and insomnia (55%), the rest comprised less than 47%. The largest improvement was reported for PTSD (83%), depression (75%), suicidality (73%), and social anxiety (71%). The remaining reported improvements from 17% (smoking) to 61%.

### Adverse effects

As shown in **Table 10**, a significant portion of participants reported no side effects from the MDMA experience (47.9%), whereas a similar portion reported some effects in the following days (42.3%). A small but notable portion reported effects from some weeks to more than a year (9.9%). The most frequent effect was sadness/dejection (30.3%), the rest were reported at lower levels (16.9% and lower, most below 10%). There were no age differences, but a complex relationship with gender appeared, suggesting that more men than women reported no adverse effects (65.3% versus 34.4%) and less effects lasting more than one year (60% versus 40%, *χ^2^* = 16,969, *df* = 8, *p* = .030). Two percent reported serious adverse effects, 4.8% sought medical help, whereas 54.8% reported no or mild effects. A majority of 57.7% reported a craving to administer the substance again, but only 5.2% reported being unsuccessful in curbing MDMA intake. Regarding acute adverse effects, 6.6% of the participants reported experiencing strong to extreme levels of fear, paranoia, bodily distress or fear of going insane as indicated by a CEQ mean score of 4–5 on at least one of the four domains.

**Table 10:** Acute and Persisting Adverse Reactions.

Reponse option	n (%)
Persistent adverse reactions after the experience	
No	291 (47.9%)
Yes, for a few days	257 (42.3%)
Yes, for a few weeks’ duration	31 (5.1%)
Yes, of a few months’ duration	14 (2.3%)
Yes, duration more than one year	15 (2.5%)
Type of persistent adverse reactions after the experience	
Nausea/vomiting	28 (4.6%)
Dizziness	36 (5.9%)
Anxiety/nervousness	103 (16.9%)
Paranoia	32 (5.3%)
Sadness/dejection	184 (30.3%)
Headache	47 (7.7%)
Difficult to concentrate	91 (15.0%)
Unrest/agitation	73 (12.0%)
Guilt	42 (6.9%)
Shame	53 (8.7%)
Irritability/anger	39 (6.4%)
Muscle tensions	57 (9.4%)
Loss of appetite	88 (14.5%)
Sleeplessness	93 (15.3%)
Hot/cold flushes or feeling	41 (6.7%)
Restlessness	63 (10.4%)
Other	62 (10.2%)
Sought professional health care to handle adverse reactions	
Yes	21 (4.8%)
No	421 (95.2%)
Perceived severity of adverse reactions	
Didn’t bother me at all	121 (19.9%)
Mild: did not affect function in everyday life	212 (34.9%)
Moderate: affected function in everyday life	69 (11.3%)
Severe: was unable to function in everyday life	12 (2.0%)
Experience of craving to administer the same substance again	
Yes	351 (57.7%)
No	257 (42.3%)
Attempt to cut the intake of MDMA without succeeding	
Yes	30 (5.2%)
No	578 (95.1%)

*N* = 608.

**Table 11** shows that the majority of participants reported a moderate, strong or extremely strong increased understanding of the significance of past life events during the MDMA experience (59.2%). A clear majority of 67.5% reported that they uncovered associations between current and past interpersonal relationships during the MDMA experience. As many as 92.7% of the participants reported that they discovered new or forgotten ways of dealing with difficulties and challenges during the MDMA experience.

**Table 11:** Attitudes and Insights. .

Response option	n (%) or M (SD)
Change in understanding of previous life events after the experience	3.67 (1.57)
Not at all	76 (12.5%)
Minimally	92 (15.1%)
Somewhat	80 (13.2%)
Moderately	148 (24.3%)
Strongly	136 (22.4%)
Extremely	76 (12.5%)
Discovery of connections between present and previous relations after the experience	4.00 (1.50)
Not at all	56 (9.2%)
Minimally	62 (10.2%)
Somewhat	80 (13.2%)
Moderately	127 (20.9%)
Strongly	195 (32.1%)
Extremely	88 (14.5%)
Discovery of forgotten or new ways of dealing with difficulties and challenges after the experience	3.57 (1.58)
Not at all	91 (15.0%)
Minimally	81 (13.3%)
Somewhat	102 (16.8%)
Moderately	124 (20.4%)
Strongly	151 (24.8%)
Extremely	59 (9.7%)

*N* = 608.

## Discussion

The present study aimed at describing the patterns of use of MDMA in Norway. More than 600 adult participants participated in an anonymous internet survey, sharing information about their most memorable experience with MDMA, including characteristics of the experience as well as adverse effects. The study was designed on the back of a similar and previously published study on classic psychedelics (28). The sub-aims were to investigate perceived change in mental disorders and substance use disorders, and adverse reactions following MDMA use, making this the second Norwegian survey study on psychedelics as a broad category. Identical questions in both surveys also make it meaningful to compare the results to examine differences in the experience of the substances.

The study sample mostly consisted of men under 35 years, a slightly less male-dominated but similar in age compared to the psychedelics study. We must assume that many participants took part in both studies, but we cannot determine how many and who they were because both surveys were anonymous. The sample participants were generally well educated and with high income, both categories at somewhat higher levels than in the psychedelics study. We could speculate that MDMA use is somewhat more prevalent among higher socio-economic status groups, whereas classic psychedelics are more prevalent among lower status groups. But we also need to caution that the sample is self-selected. Studies targeting the general population, such as (26, 27, 37), have shown a more mixed pattern of users and not the least more problematic use than in our sample. Our study is best suited to cast light on what characterizes a more resourceful and dedicated group of MDMA users.

### Patterns of recreational MDMA use

Most participants reported on a MDMA only experience, some combined MDMA with alcohol, and some also with other substances. Overall frequency of use was low in the sense that the highest frequency category was two to five occasions both last year and lifetime use. Still, 68.6% of participants reported MDMA use within the past 12 months, and over 60% reported more than five experiences, meaning that we had an experienced sample. This finding contrasts sharply with the data published in 2024 by the Norwegian Institute of Public Health, which indicated that only 1.4% of Norwegians aged 16–64 and 3.2% of those aged 16–30 had used MDMA the past 12 months (4). This large difference is likely a reflection of the convenience sampling resulting in a non-representative sample with more consistent and dedicated users, where the national survey used a random draw from a large representative sample of Norwegians (4).

### Characteristics of the most memorable MDMA experience

Notable distinctions exist between the administration of MDMA in clinical versus nonclinical settings, particularly regarding preparation before, support during and integration following the MDMA experience (38). But there are also similarities in the way our participants approached and structured the experience. Most participants in our survey took a moderate or high dose, and they recalled the experience well. They were generally well-prepared and approached it with clear intentions, indicating that they aimed for a “peak” experience intended to increase well-being (39). However, only a subset reported explicit therapeutic motivations, and both the reported desire for change and post-experience integration work were limited in the sample. Still, about half of the participants received guidance or support, and many reported gaining deeper insights into life events and relational patterns, as well as strategies for coping with challenges. The participants also held positive attitudes toward MDMA’s therapeutic potential. We are therefore wondering whether the label “therapeutic” is too closely tied to having mental disorders and have therefore not felt appropriate for many participants. The connection between well-defined set and setting on the one hand, including expectancy effects, and positive and negative outcomes on the other, needs further exploration. For now, we conclude that while there were some similarities, key differences also emerged between the set and setting in this survey compared to MDMA-assisted therapy in clinical trials.

### Perceived change in mental disorders, substance use disorders, and personality

Participants in our survey reported substantial improvements in PTSD following their memorable MDMA experience. This is consistent with the Phase 3 trials of MDMA-AT for PTSD (9, 10), which demonstrated a significant reduction in symptoms in the MDMA group compared to the placebo group. Notably, our participants also reported significant improvements in depression, both in the sample as a whole and in a subset with depression (40). One of the Phase 3 trials of MDMA-AT for PTSD found a statistically significant reduction in depression symptoms (9), and the first proof-of-principle study on MDMA-AT for major depressive disorder was recently completed in Norway (41). Interestingly, the three conditions with the greatest improvements in this survey—PTSD, depression, and suicidality—mirror exactly those in our previous survey on classic psychedelics (28). The reason for this remains unclear, though overlap between study populations may be a factor.

The explanations for the corresponding results can be manifold. One could be that the drug targets specific neural and psychological mechanisms that lead to improvements in the conditions. Another could be that our sample has to a large degree applied the same therapeutic mindset and framing that also characterizes clinical studies. Most likely we are seeing a combination of the two.

The results on personality changes following administration of psilocybin are mixed (42), but on MDMA research is mostly lacking all together. A comparison of personality traits between groups of “hard” and “non-hard” drug users found differences in trait composition but did not examine effects on personality traits following MDMA use (43). Wagner et al. (44) found an increase in openness and a decrease in neuroticism in connection with PTSD treatment, whereas a meta-analysis found increased openness, but also more adverse effects connected to higher neuroticism (11). We measured levels of personality functioning, which is connected to higher neuroticism, but is a more general measure of overall functioning. Our results indicate that recreational MDMA use may be connected to improved overall functioning and therefore better mental health. The connection between level of personality functioning, diagnoses, and well-being is complex and needs further exploration, but we want to argue that we should move beyond symptom load and categorical diagnoses when measuring possible beneficial effects of MDMA. Our results may also inform future research on MDMA-assisted personality disorder treatment (45).

### Adverse effects

Our participants most frequently reported adverse effects were sadness/dejection (30.3%), anxiety/nervousness (16.9%), sleeplessness (15.3%) and difficulties concentrating (15%). This further supports the evidence related to negative effects on mood and cognition in the days following dosing, also known as ‘Blue Mondays’. This has been found for recreational MDMA users (23) and in participants in controlled trials (22, 24). Overall, our participants reported relatively few and quite short-lived adverse effects, and very few reported long-lasting adverse effects. This result is in line with at least one previous survey on recreational MDMA use (5). We would also like to note that participants in the classic psychedelics study reported significantly higher levels of psychological challenges on the CEQ than our participants did (5.99 versus 2,56; 28). MDMA experiences therefore seem to be far less challenging than experiences with psychedelics.

The observed low degree of challenging experiences may be, in part, a function of our sampling methodology. It is plausible that individuals who had particularly negative or traumatic experiences with MDMA were less inclined to participate in a survey of this nature, leading to a potential self-selection bias. It is also important to consider that the concept of a “meaningful experience” does not preclude it from being psychologically challenging, and that difficult experiences can often be a crucial component of a therapeutic process. Furthermore, the high reported rates of preparation and integration work within our sample may reflect a greater awareness of harm-reduction practices, which itself could be a characteristic of the self-selecting population. Consequently, our findings may be more indicative of the MDMA experience when it is approached with a conscientious focus on set and setting, rather than a representation of a more general population. This perspective, however, provides valuable insights into how therapeutic benefits might be optimized in non-clinical contexts.

### Limitations

The present study has several limitations that should be noted. First of all, a cross-sectional study like ours is not suited as a basis for causal conclusions. Second, the retrospective design makes the data susceptible to recall bias. Most participants reported that they remember the experience well, but we do not know how accurate this recall is. A substantial majority reported use of MDMA on several occasions, and it can therefore be hard to separate negative and positive effects experienced in other sessions from the single most memorable experience. Third, a response bias is to be expected, both because of the self-report format and because of participant selection. Self-report is susceptible to socially desirable responding influenced by media coverage, peer influence (we cannot know if participants filled in the form alone), from various degrees of prior knowledge of the subject, and more. Especially regarding self-reported diagnoses that are not reported as set by a clinician, we need to consider the possibility that participants feel they have a diagnosis although the criteria may not be fulfilled. In addition, we should expect that we have an overrepresentation of responders with positive experiences and attitudes. Given that we recruited from web sites that provide neutral information about drugs and drug use, we may also have a selection of participants that are above average in knowledge and curiosity than a representative sample would be. Those with negative, and perhaps only negative experiences with MDMA, are less likely to participate as they might have shunned the whole topic. Furthermore, our participant sample may not be representative for the average MDMA user, especially in terms of its high level of education and income. Finally, and as a summary of the previous points, more research is needed into less privileged and less positively inclined participants to get a more complete picture of the possible negative effects of recreational MDMA use.

## Conclusion

Despite the limitations inherent in a self/selection study, this anonymous internet survey is an important addition to the knowledge base about risks and benefits from MDMA experiences in a recreational setting. Most of the participants used MDMA for recreational and therapeutic purposes, were well prepared before the experience and did integration work afterwards. Mental disorders and substance use disorders were prevalent in the sample, and most participants reported improvements in their condition(s). Adverse reactions were mostly tolerable and transient. However, a small subset of participants experienced persistent adverse reactions for more than a year.

## Data Availability

The datasets presented in this article are not readily available because we used a secure web application for data storage (Services for Sensitive Data; TSD), which meets requirements for the processing and storage of sensitive research data. Access to the dataset requires special permission, and could be available on reasonable request to the first author. Requests to access the datasets should be directed to CG, cato.gronnerod@psykomatikk.no.
